# Autaptic regulation of electrical activities in neuron under electromagnetic induction

**DOI:** 10.1038/srep43452

**Published:** 2017-02-27

**Authors:** Ying Xu, Heping Ying, Ya Jia, Jun Ma, Tasawar Hayat

**Affiliations:** 1 Department of Physics, Lanzhou University of Technology, Lanzhou 730050, China; 2 Institute of Biophysics and Department of Physics, Central China Normal University, Wuhan 430079, China; 3 Department of Physics, Zhejiang University, Hangzhou 310027, China; 4 King Abdulaziz Univ, Fac Sci, Dept Math, NAAM Res Grp, Jeddah 21589, Saudi Arabia; 5 Quaid I Azam Univ, Dept Math, Islamabad 44000, Pakistan

## Abstract

Realistic neurons may hold complex anatomical structure, for example, autapse connection to some internuncial neurons, which this specific synapse can connect to its body via a close loop. Continuous exchanges of charged ions across the membrane can induce complex distribution fluctuation of intracellular and extracellular charged ions of cell, and a time-varying electromagnetic field is set to modulate the membrane potential of neuron. In this paper, an autapse-modulated neuron model is presented and the effect of electromagnetic induction is considered by using magnetic flux. Bifurcation analysis and sampled time series for membrane potentials are calculated to investigate the mode transition in electrical activities and the biological function of autapse connection is discussed. Furthermore, the Gaussian white noise and electromagnetic radiation are considered on the improved neuron model, it is found appropriate setting and selection for feedback gain and time delay in autapse can suppress the bursting in neuronal behaviors. It indicates the formation of autapse can enhance the self-adaption of neuron so that appropriate response to external forcing can be selected, this biological function is helpful for encoding and signal propagation of neurons. It can be useful for investigation about collective behaviors in neuronal networks exposed to electromagnetic radiation.

Since the pioneering experimental work^1^ about electrical activities on large axon of squid, many neuron models^2^,^3^,^4^,^5^,^6^,^7^ provide available ways to investigate the dynamical properties of electrical activities in neuron and even neuronal networks. As a result, bifurcation parameters and noise are changed to detect mode transition in electrical activities. On the other hand, stochastic resonance, coherence resonance, synchronization between neurons and neuronal networks have been investigated extensively^8^,^9^,^10^,^11^,^12^,^13^,^14^,^15^,^16^,^17^,^18^. It is believed that synapse^19^ is important to bridge the neurons for signal exchange and communication. Autapse, a specific synapse which connects the its own body of neuron via close loop^20^,^21^, and it is confirmed that autaptic driving and autapse connection to neuron can regulate the electrical behavior of neuron greatly^22^,^23^,^24^,^25^,^26^,^27^,^28^,^29^,^30^. For example, autapse driving can induce synchronization and regular pattern formation in the neuronal network by generating continuous wave front like a pacemaker. Particularly, autapse modulation can be effective to induce coherence resonance^31^ and further used for signal detection based on stochastic resonance^32^. Besides these theoretical neuron models, some researchers have designed effective neuronal circuits^33^,^34^,^35^,^36^,^37^,^38^ to reproduce the electrical activities such as quiescent, spiking, bursting and even chaotic states, respectively. For a brief introduction in the field of neurodynamics, readers can refer to refs 39, 40 and 41 and the references therein.

Both of the deterministic and stochastic neuron models can produce the main dynamical properties in electrical activities of neuron, and bifurcation parameters can be modulated to induce transition of modes, pattern stability, and synchronization transition of neuronal networks even noise is considered. In fact, another important factor should be considered that the fluctuation of concentration distribution of charged ions of cell can induce time-varying electromagnetic field in the cell, as a result, the exchange of ions across the membrane and membrane potential can be modulated by induced currents. Therefore, the author of this paper suggested that magnetic flux^42^ can be used to describe the effect of electromagnetic field and also memristor is used to realize coupling between membrane potential and magnetic flux. The improved model^43^,^44^ can be effective to detect the effect of electromagnetic radiation by applying external magnetic flux associated with electromagnetic field on the dynamical equation for magnetic flux. It is found that external electromagnetic radiation can induce multiple modes^45^ in electrical activities. It is thought that autapse connection to neuron can play active role in regulating the electrical activities, and could make neuron become self-adaptive and give appropriate response to external stimuli, thus appropriate modes in electrical activities can be selected. Therefore, it is interesting to investigate the same problem when electromagnetic induction and radiation, and noise are considered on the neuron. In this paper, autaptic modulation and electromagnetic radiation are imposed on the improved neuron, the mode selection and transition, Hamilton energy^46^,^47^ will be calculated, respectively. The possible biological function of autapse connection will be discussed.

## Model, Method and Proof

The original Hindmarsh-Rose neuron model is produced from the biological Hodgkin-Huxley neuron model, and it is available for bifurcation analysis in electrical activities in neuron. According to the physical law of electromagnetic induction, continuous exchange of charged ions across the membrane can trigger complex fluctuation in ion concentration and set up time-varying electromagnetic field to adjust the membrane potential of neurons. Indeed, magnetic flux across the membrane can fit this requirement, thus magnetic flux *φ* is used as additive variable to describe the effect of electromagnetic induction and field. Furthermore, to be consistent with physical unit, memristor is used to realize coupling on membrane potential of neuron, the memductance of memristor^42^ is often described by


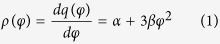


where *φ* is the magnetic flux across the memristor, *α, β* are parameters, *q* is the charge across the memristor. The improved neuron driven by autapse can be described as follows


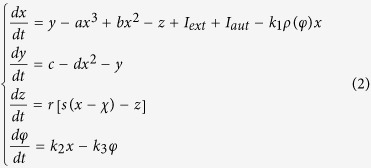


where *x, y, z* is the membrane potential, recovery variable for slow current and adaption current, respectively. *I*_*ext*_ is external forcing current, the term *k*_1_*ρ(φ)x* describes the induced current induced by electromagnetic induction, the physical unit is verified as follows


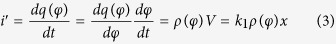


where *V* is the induced voltage and holds the same physical units as variable *x,* and the parameter *k*_1_ describes the modulation intensity of induced current on the membrane potential. The memductance of memristor is described by *ρ(φ*) = *α* + 3*βφ*^2^, the parameters are often set as *a* = 1.0, *b* = 3.0, *c* = 1.0, *d* = 5, *s* = 4.0, *r* = 0.006, *χ* = −1.56, *α* = 0.4, *β* = 0.02, *k*_1_ = 0.4, *k*_2_ = 0.9, *k*_3_ = 0.5. Similar to the previous model without magnetic flux and field being considered, different modes in electrical activities can be selected by applying appropriate forcing current on the neuron. Indeed, the autaptic current from the autapse can also play important role in regulating the electrical activities and even the excitability of neuron, the electric autapse current is given by


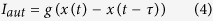


where *g, τ* is the feedback gain and time delay in the autapse loop. A positive setting for *g* can produce positive feedback and excite the quiescent neuron while negative gain *g* will induce negative feedback on the membrane and can calm down the electrical activities in neuron. In the following, the effect of autapse driving on the neuronal activities will be investigated on the new neuron model with electromagnetic induction being considered. At first, appropriate external forcing current is set to trigger bursting state and then autapse driving is switched to regulate the electrical activities thus the biological function of autapse connection with different time delay and feedback gains can be understood.

As it is well known, the emergence of action potential and mode transition in electrical activities are dependent on the energy consumption and supply. It is suggested that Hamilton energy can be estimated by Helmholtz theorem^48^ that the general vector function of position can be written as a gradient plus a curl as follows


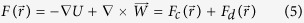


where *U* is a scalar function and ***W*** is a vector function and the minus sign in front of *∇U* is a convention. It often reads^47^


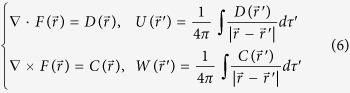


in which the integrals are over all space. As reported in refs 46, 47 and 49, a general Hamilton energy function *H* can be defined by the following criterion


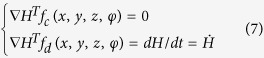


As a result, the dynamical system described by Eq. (2) can be rewritten by


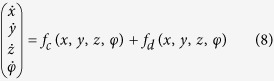


And it reads as follows


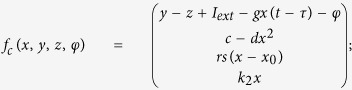



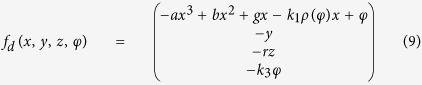


Therefore, the Hamilton energy can be estimated according to the criterion in Eq. (7), and it finds


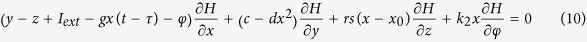


Indeed, an appropriate solution can be approached for Eq. (10) thus the Hamilton energy is detected by


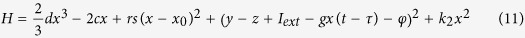


Furthermore, it can be verified as follows


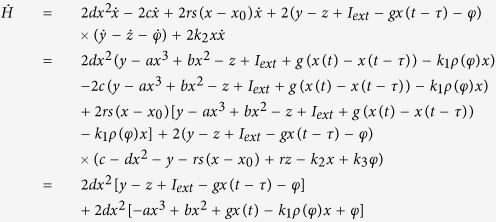



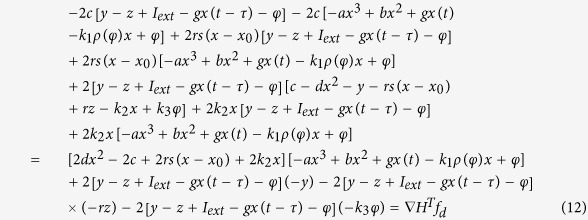


That is to say, the Hamilton energy is dependent on all the variables (voltage and current) in Eq. (2) except the magnetic flux, the electrical field energy is associated with membrane potential and current, and the magnetic field energy is associated with induced current. In the following, the energy release will be calculated during the control and transition of mode in electrical activities when autapse driving, electromagnetic radiation and noise are considered.

## Numerical Results and Discussion

**Figure 1:**
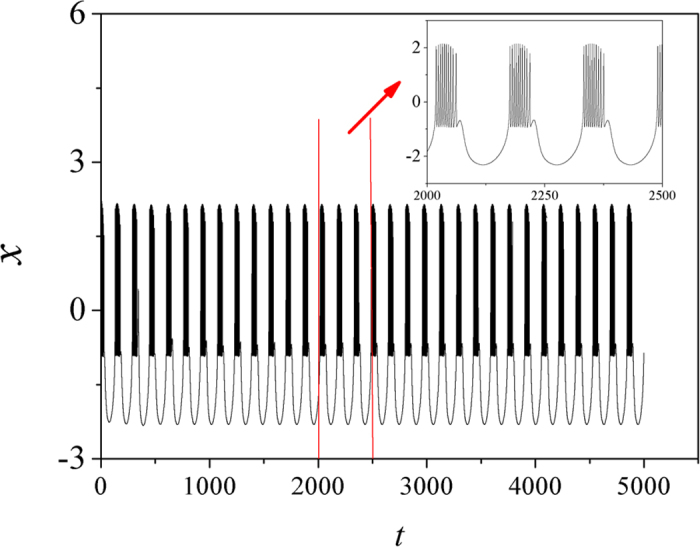
Sampled time series for membrane potential of neuron are calculated when external forcing current is set as *I*_*ext*_ = cos (0.02*t*), no autapse driving is considered.

In the numerical investigation, the fourth Runge-Kutta algorithm is applied to find solutions for the delayed dynamical system with time step *h* = 0.01, the initials for variables (*x, y, z, φ*) are set as (0.3, 0.1, 0.2, 0), the transient period for calculating is about 5000 time units, the autapse driving begins to regulate the membrane potential along the autapse loop at *t* = 1000 time units. At first, the external forcing current is set as *I*_*ext*_ = cos(0.02*t*) and the dynamical response of electrical activities is calculated in Fig. 1.

**Figure 2:**
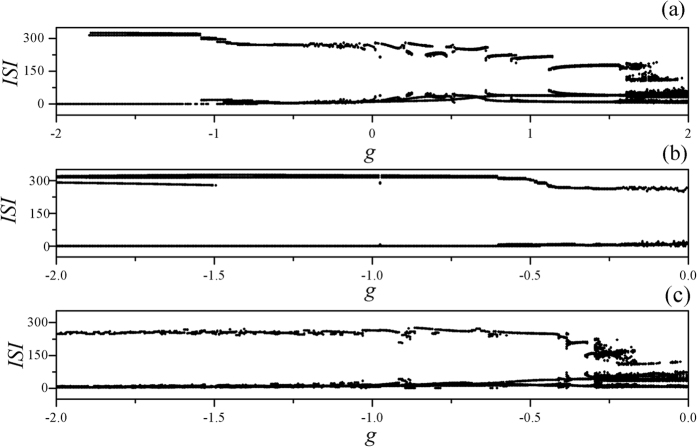
Bifurcation diagram for ISI is calculated by changing the feedback gain in autapse. The time delay is fixed at *τ* = 20 (**a**), *τ* = 10 (**b**) with electromagnetic induction being considered, for *τ* = 20 (**c**) without electromagnetic induction, the external forcing current is set as *I*_*ext*_ = cos (0.02*t*).

It is found in Fig. 1 that the neuronal activities present bursting type when periodical forcing is applied on the neuron. It shows some difference from the original three-variable Hindmarsh-Rose neuron that periodical discharging can be induced by periodical forcing current. To discern the effect of electric autapse driving, the autaptic current is triggered at *t* = 1000 time units. At first, the time delay *τ* is fixed, the bifurcation diagram of ISI (interspike interval) is calculated by setting different feedback gains *g*, and the results are plotted in Fig. 2.

**Figure 3:**
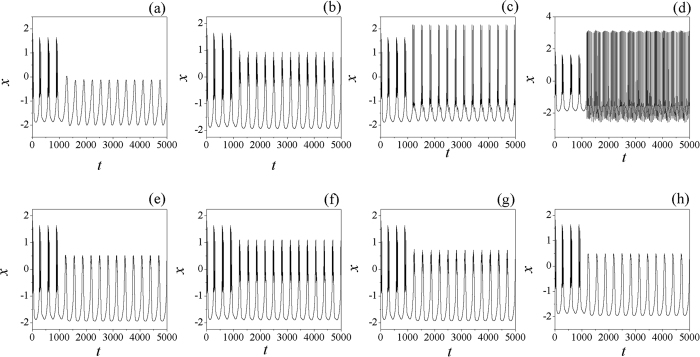
Sampled time series for membrane potential are calculated under different feedback gains. In the first layer of the panel, the time delay is fixed *τ* = 20, for (**a**) *g* = −2.0, (**b**) *g* = −0.5, (**c**) *g* = 0.5, (**d**) *g* = 2.0. In the second layer, the time delay is fixed *τ* = 10, for (**e**) *g* = −0.5,(**f**) *g* = −0.9, (**g**) *g* = −1.0, (**h**) *g* = −1.1. The external forcing current is set as *I*_*ext*_ = cos (0.02*t*).

The bifurcation diagram in Fig. 2 found that the mode of electrical activities is much dependent on the selection of feedback gain and also the time delay in autapse, it is some different from the case without electromagnetic induction being considered. That is to say, the mode of electrical activities in neuron become more complex when both of autapse driving and electromagnetic induction (intrinsic field) are considered altogether. Furthermore, the sampled time series are recorded to explore the dynamical properties of electrical activities of neuron driven by autapse when electromagnetic induction is considered, the results are found in Fig. 3.

**Figure 4:**
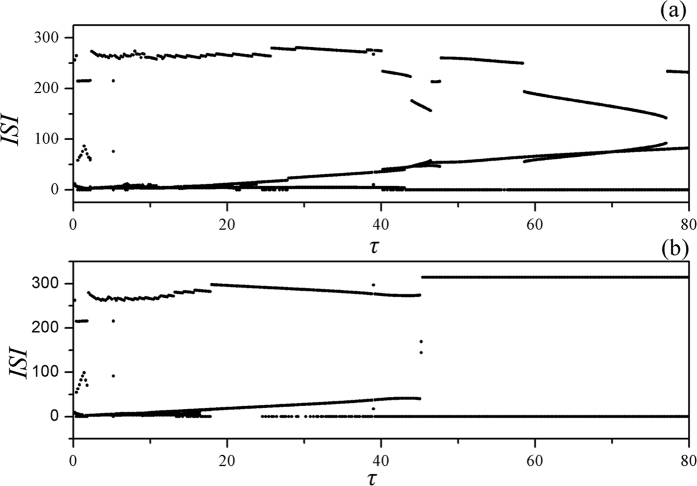
Bifurcation diagram for ISI is calculated by changing the time delay in autapse. The feedback gin is fixed at *g* = −0.7(**a**), *g* = −1.0 (**b**), the external forcing current is set as *I*_*ext*_ = cos (0.02*t*).

The results in Fig. 3 confirmed that negative feedback can suppress the electrical activities while positive feedback can enhance the discharge and bursting occurrence in electrical activities. It also shows some difference from the case when electromagnetic induction is not considered. The potential mechanism could be that energy storage in electromagnetic field make neuron keep against negative feedback, otherwise, the neuron will be suppressed into quiescent state under negative feedback. In the presence of external forcing current, positive feedback in autapse make neuron supply more energy and can be saved in electromagnetic field, thus the electrical activities become more complex in rhythm. It is found that time delay in autapse also plays important role in changing the electrical activities when electromagnetic induction is considered, and bursting electrical activities will be suppressed into spiking by increasing the negative feedback gain in autapse. In fact, the intrinsic time delay in neuron such as time delay in response and autapse loop can be finite, so it is important to find detailed effect of time delay on the electrical activities in neuron, the results are plotted in Fig. 4.

The calculation for ISI in Fig. 4a confirmed that mode in electrical activities can be changed by setting appropriate time delay even when the feedback gain in autapse is fixed at negative value, and longer time delay in autapse can be available for suppressing the bursting behavior, the amplitude and discharge rhythm can be suppressed to develop spiking state. Furthermore, the negative feedback is enhanced to investigate the response of neuronal activities under autapse driving, the bifurcation analysis is presented in Fig. 4b, and the bursting state can be more effective to be suppressed. Indeed, the bursting state is removed by applying longer time delay in autapse with increasing the negative feedback gain, and the discharge rhythm will be stabilized when the time delay is beyond certain threshold about 45.

**Figure 5:**
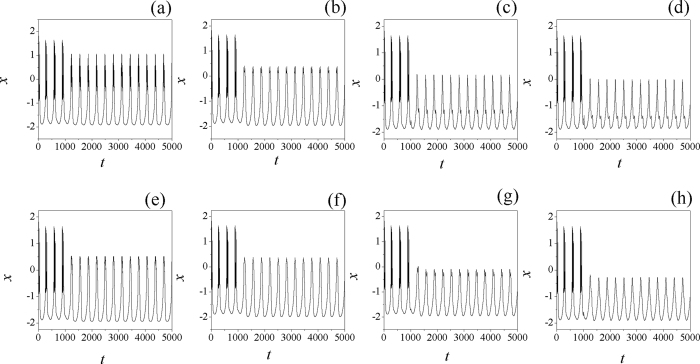
Sampled time series for membrane potential are calculated at different time delays. In the first layer of the panel, feedback gain *g* = −0.7, for time delay (**a**) *τ* = 10, (**b**) *τ* = 30, (**c**) *τ* = 60, (**d**) *τ* = 80. In the second layer of panel, feedback gain *g* = −1.0, for time delay (**e**) *τ* = 10, (**f**) *τ* = 20, (**g**) *τ* = 40, (**h**) *τ* = 50. The external forcing current is set as *I*_*ext*_ = cos (0.02*t*).

Furthermore, the sampled time series are detected to illustrate the changes of membrane potential of neuron driven by electric autapse with different time delay being considered, the results are shown in Fig. 5.

**Figure 6:**
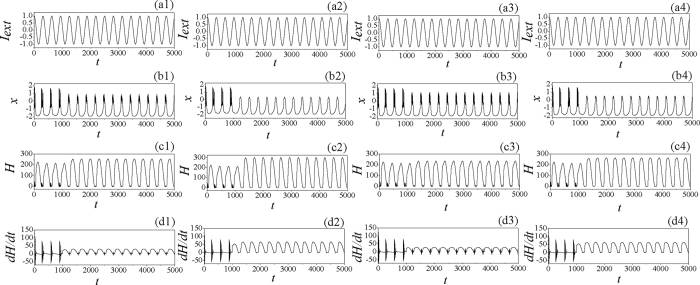
Response of electrical activities, energy function are calculated when neuron is driven by autapse. For (a1,b1,c1,d1) *g* = −0.5, *τ* = 20; (a2,b2,c2,d2) *g* = −1.0, *τ* = 20; (a3,b3,c3,d3) *g* = −0.5, *τ* = 10; (a4,b4,c4,d4) *g* = −1.0, *τ* = 10. The autaptic driving is switched on at *t* = 1000 time units.

It is found in Fig. 5 that the previous bursting state can be suppressed by further increasing the time delay in autapse when the negative feedback gain in autapse is weak. It is found that negative feedback at *g* = −0.7 is effective to suppress the bursting states, and the electrical activities used to select spiking than periodical oscillating even the neuron is driven by periodical forcing current. The potential mechanism could be that neuron can encode the external forcing and give appropriate response to external forcing. As mentioned above, energy consumption is associated with selection of electrical activities, then the Hamilton energy is calculated according to Eq. (11), the results are plotted in Fig. 6 with different condition being applied.

It is confirmed that periodical forcing generates bursting behavior in neuron, and the mode of electrical activities is changed by autaptic modulation thus bursting behavior is suppressed by negative feedback in autapse. The Hamilton energy is decreased under bursting behavior and periodical discharge can keep higher energy level. By decreasing the feedback gain(more negative), it is more effective to suppress the bursting behavior and the Hamilton energy is increased. The potential mechanism is that bursting behavior can release energy quickly and thus the energy is decreased, as a result, transition from bursting behavior to spiking state can increase the Hamilton energy when neuron is driven by autapse with negative feedback.

It is found that distinct bursting behaviors can be triggered in the time series for membrane potential by periodical forcing, then bursting behaviors can be suppressed by negative feedback from autapse. Mode transition of electrical activities often induce transition in Hamilton energy, and bursting state make the Hamilton energy become smaller, the potential mechanism could be energy can be released rapidly during the emergence of bursting state.

**Figure 7:**
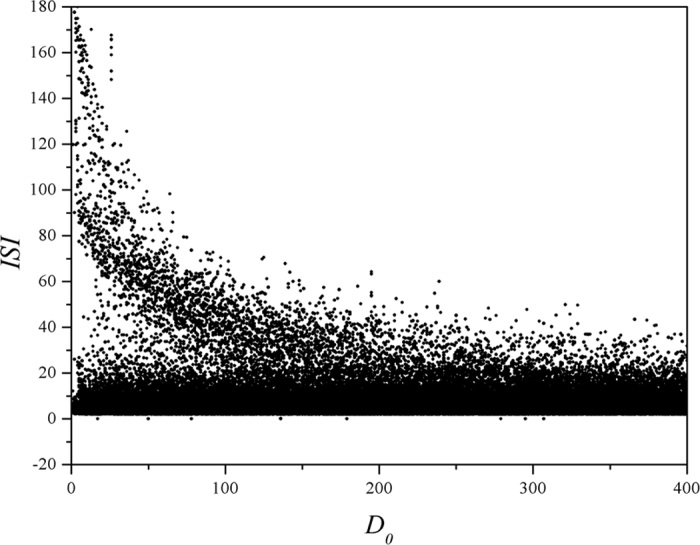
Bifurcation diagram is calculated *vs*. intensity of noise when the feedback gain and time delay in autapse are set as *g* = *−*2, τ = 20. Noise is imposed on the neuron from *t* = 0, autapse driving is switched on at *t* = 1000 time units, ISI is calculated from *t* = 2000 to 5000 time units.

It is believed that noise can change the dynamical properties in electrical activities, while autapse driving makes the neuronal become self-adaptive response to electrical forcing. For simplicity, noise is imposed on the neuron to detect the possible mode transition in electrical activities and suppression of autaptic modulation of autapse connected to neuron, it reads


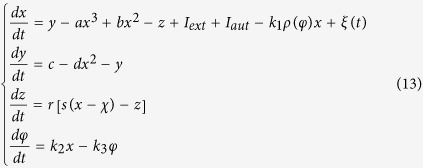


where *ξ(t*) is Gaussian white noise, and its statistical properties can be defined as 〈*ξ(t*)〉 = 0 and 〈*ξ(t)ξ(t*′)〉 = 2*D*_0_*δ(t* *−* *t*′), *D*_0_ is the noise intensity. At first, the bifurcation analysis is carried at fixed feedback gain in autapse by changing the noise intensity, the results are plotted in Fig. 7.

**Figure 8:**
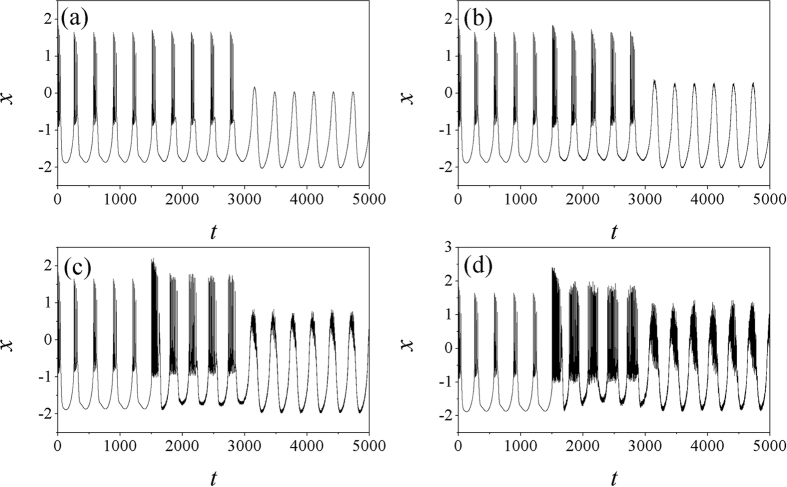
Sampled time series for membrane potential are calculated, noise is triggered at *t* = 1500 time units, for noise intensity (**a**) *D*_0_ = 1, (**b**) *D*_0_ = 10, (**c**) *D*_0_ = 100, (**d**) *D*_0_ = 300, autapse driving is switched on at *t* = 3000 time units with feedback gain g = *−*2 and *τ* = 20.

It is found that ISI shows certain diversity at fixed feedback gain in autapse which it means that bursting state can be triggered under noise, while the value for ISI is suppressed with increase of the noise intensity, it could indicate transition from bursting to periodical discharge and also accompanied by certain bursting as well. Furthermore, sampled time series are calculated under different intensities of noise, and the results are plotted in Fig. 8.

**Figure 9:**
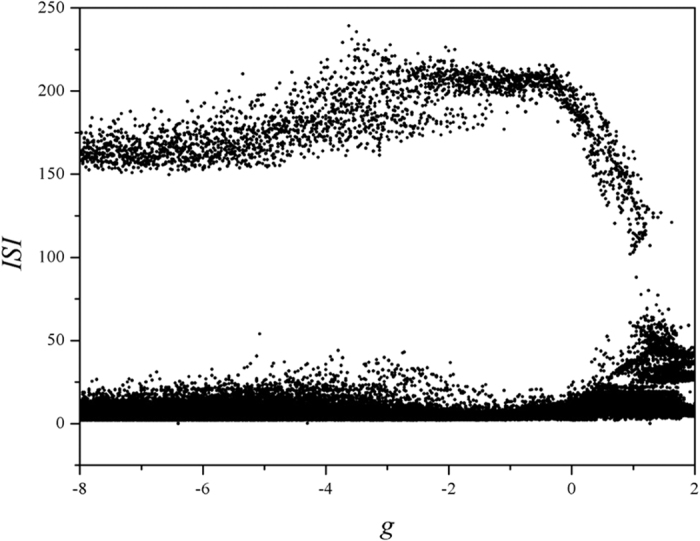
Bifurcation diagram is calculated vs feedback gain in autapse, the time delay is fixed *τ* = 20 and noise intensity *D*_0_ = 100. Noise is imposed on the neuron from *t* = 0, autapse driving is switched on at *t* = 1000 time units, ISI is calculated from *t* = 2000 to 5000 time units.

**Figure 10:**
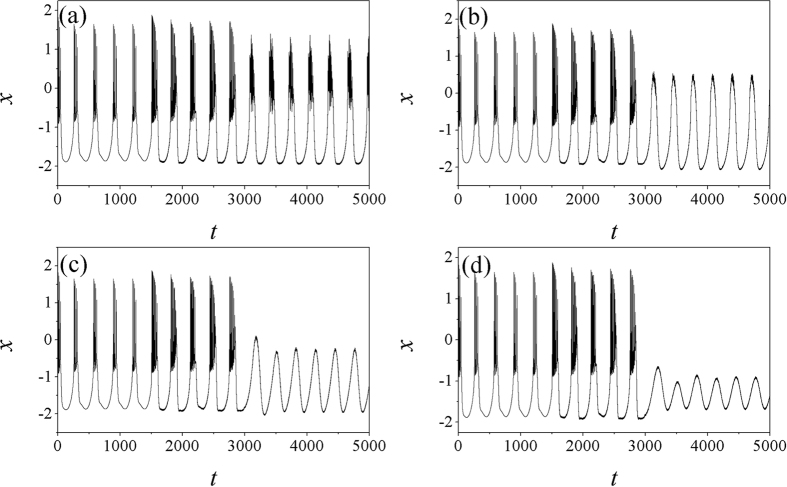
Sampled time series for membrane potential are calculated, noise is triggered at *t* = 1500 time units, for noise intensity *D*_0_ = 100 and time delay *τ* = 20, autapse driving is switched on at *t* = 3500 time units with feedback gain for (**a**) *g* = 1, (**b**) *g* = −1, (**c**) *g* = −4, (**d**) *g* = −8.

It is found in Fig. 8 that the bursting behaviors can be enhanced with increasing the noise intensity, and the autapse driving can suppress the bursting behavior before the noise intensity is increased greatly. That is to say, autaptic modulation with negative feedback can remove the bursting state and develop stable periodical discharge when the noise intensity is intermediate. Otherwise, the electrical activities keep bursting with increasing the noise intensity. Furthermore, the effect of feedback gain in autapse is investigated in presence of noise, and the bifurcation analysis is presented in Fig. 9, and sampled time series are plotted in Fig. 10.

The results in Fig. 9 confirmed that electrical activities can be effectively suppressed by autapse driving with negative feedback while positive feedback can enhance bursting behaviors. Moderate feedback gain in autapse can trigger mixed modes in electrical activities that bursting is hidden in periodical discharge, and sampled time series are calculated in Fig. 10.

**Figure 11:**
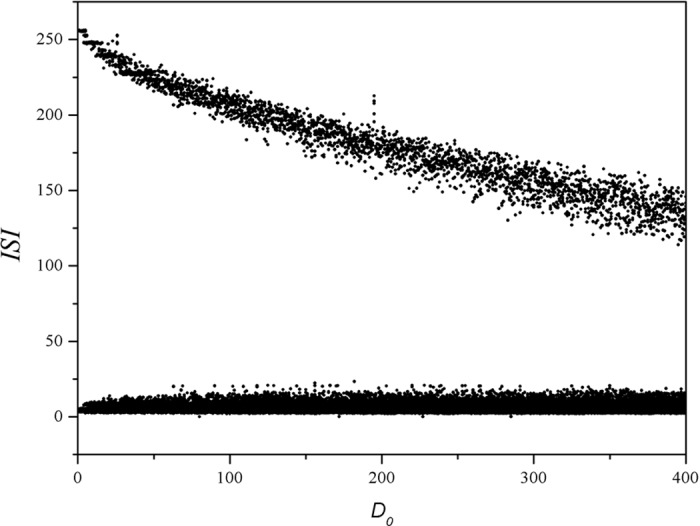
Bifurcation diagram is calculated vs noise intensity, the time delay and feedback gain is fixed at *g* = *−*0.5 and τ = 20, respectively. Noise is imposed on the neuron from *t* = 0, autapse driving is switched on at *t* = 1000 time units, ISI is calculated from *t* = 2000 to 5000 time units.

**Figure 12:**
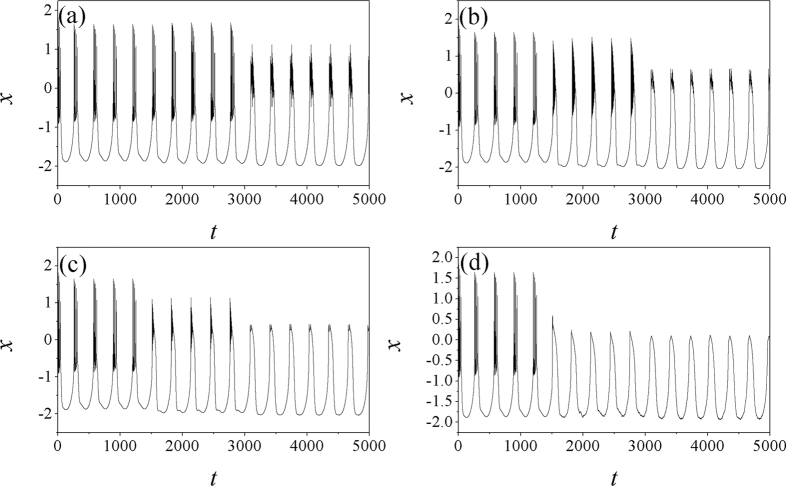
Sampled time series for membrane potential are calculated, noise is triggered at *t* = 1500 time units, for noise intensity (**a**) *D*_0_ = 10, (**b**) *D*_0_ = 100, (**c**) *D*_0_ = 200, (**d**) *D*_0_ = 500, autapse driving is switched on at *t* = 3000 time units with feedback gain *g* = −0.5 and *τ* = 20.

Indeed, positive feedback in autapse can enhance the bursting state, while autaptic driving also can suppress the electrical activities under negative feedback with appropriate gain being used and the modulation shows certain robustness to noise. It is also important to investigate the case when noise-like electromagnetic radiation is imposed on the network, it reads


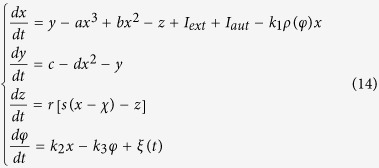


where *ξ(t*) is Gaussian white noise and holds the same statistical properties in Eq. (13). The noise is set under different intensities and the bifurcation analysis is presented in Fig. 11, and sampled time series are plotted in Fig. 12.

There are some differences between the bifurcation diagram in Figs 11 and 7, the potential mechanism could be that noise-like input is imposed on neuron with different ways (or channels). In Figs 7 and 8, the membrane potential is changed directly by applying external forcing current with Gaussian type of distribution in time, while Gaussian noise-like electromagnetic field in Fig. 11 is imposed on neuron thus the magnetic flux is changed to further modulate the dynamical behaviors in electrical activities. Furthermore, the sampled time series are calculated in Fig. 12 with autapse driving being considered as well.

In fact, stochastic inputting of electromagnetic field is also effective to change the electrical activities by applying noise-like magnetic flux on neuron, the potential mechanism could be that the neuronal activities is changed by induced current. Furthermore, a negative feedback gain in autapse become more effective to suppress the bursting behavior with increasing the intensity of external electromagnetic radiation.

In a summary, in the presence of electromagnetic induction, autapse driving also plays important role in regulating the dynamical behaviors of neuronal activities. For example, bursting state is suppressed by negative feedback while bursting state is enhanced under positive feedback in autapse. That is, the formation of autapse can enhance the self-adaption of neuron exposed to external electromagnetic radiation thus appropriate response in electrical activities can be selected for normal signal encoding and propagation.

## Conclusions

In this paper, the possible biological function of autapse connection to neuron is investigated when the effect of electromagnetic induction is considered. Autapse can switch between negative feedback and positive feedback so that the neuron can give appropriate response to external forcing. For example, positive feedback in autapse can enhance the discharge and bursting behaviors, while negative feedback in autapse can suppress the oscillating behaviors and generate periodical spiking with appropriate rhythm. These results confirmed the self-adaption of neuron by developing appropriate autapse connection even electromagnetic induction and even radiation are under consideration.

## Additional Information

**How to cite this article**: Xu, Y. *et al*. Autaptic regulation of electrical activities in neuron under electromagnetic induction. *Sci. Rep.* **7**, 43452; doi: 10.1038/srep43452 (2017).

**Publisher's note:** Springer Nature remains neutral with regard to jurisdictional claims in published maps and institutional affiliations.
